# Improvement of *Echinacea purpurea* and *Ganoderma lucidum* Extracts with Cell Model on Influenza A/B Infection

**DOI:** 10.3390/molecules29153609

**Published:** 2024-07-30

**Authors:** Bo-Kai Chen, Chi-Ho Chan, Arthur Tsao, Chin-Kun Wang

**Affiliations:** 1 Department of Nutrition, Chung Shan Medical University, 110, Section 1, Jianguo North Road, Taichung 40201, Taiwan; gargon88@gmail.com; 2 Department of Microbiology and Immunology, Chung Shan Medical University, Taichung 40201, Taiwan; chiho@csmu.edu.tw; 3 Department of Medical Research, Chung Shan Medical University Hospital, Taichung 40201, Taiwan; 4 SFG Health Lab, Standard Foods Group, Taoyuan 337402, Taiwan; arthurtsao@sfworldwide.com

**Keywords:** *Echinacea purpurea*, *Ganoderma lucidum*, influenza virus, mitochondria

## Abstract

Since 2019, COVID-19 has been raging around the world. Respiratory viral infectious diseases such as influenza and respiratory syncytial virus (RSV) infection are also prevalent, with influenza having the ability to cause seasonal pandemics. While vaccines and antiviral drugs are available to prevent and treat disease, herbal extracts would be another option. This study investigated the inhibitory effects of extracts of *Echinacea purpurea* (EP) and *Ganoderma lucidum* (*G. lucidum*) and the advanced *G. lucidum* drink (AG) on influenza A/B viruses. To determine whether EP and *G. lucidum* extracts enhance cell immunity and thus prevent virus infection or act to directly suppress viruses, cell survival and hemagglutination (HA) assays were used in this study. Cells were treated with samples at different concentrations (each sample concentration was tested from the highest non-cytotoxic concentration) and incubated with influenza A/B for 24 h, with the results showing that both *G. lucidum* and EP extracts and mixtures exhibited the ability to enhance cell survival against viruses. In the HA assay, AG and EP extract showed good inhibitory effect on influenza A/B viruses. All of the samples demonstrated an improvement of the mitochondrial membrane potential and improved resistance to influenza A/B virus infection. EP and *G. lucidum* extracts at noncytotoxic concentrations increased cell viability, but only AG and EP extract directly decreased influenza virus titers. In conclusion, results indicate the ability of EP and *G. lucidum* extract to prevent viruses from entering cells by improving cell viability and mitochondrial dysfunction and EP extract showed direct inhibition on viruses and prevented viral infection at post-infection strategy.

## 1. Introduction

Influenza viruses belong to the family *Orthomyxoviridae*. The influenza virus is a negative-sense single-stranded RNA virus with eight RNA gene segments, which can cause severe respiratory diseases. The viral genome encodes the surface glycoproteins hemagglutinin (HA) and neuraminidase (NA). According to their matrix protein (M) and nucleoprotein (NP), antigenicity can be divided into three genera: A, B and C [1]. HA enables the virus to attach to cell surface receptors and internalize, while NA is required for the release of virions from infected cells. Influenza viruses are primarily in airway epithelial cells, leading to inflammation, congestion of the airways, and tissue necrosis. The disease manifests as upper and lower respiratory tract involvement [2]. Influenza is the most common viral infectious disease of the upper respiratory tract in children, adolescents, and the elderly. Influenza usually begins in autumn and winter every year and then spreads rapidly in winter, but in southeast Asia and Taiwan, influenza cases also occur in summer [3,4].

*Echinacea purpurea* (EP) is a native purple coneflower used in North America to treat various infectious diseases and wounds [5]. It is a medicinal plant with important immunostimulatory properties [6]. Previous studies have shown that EP has immunomodulatory, anti-inflammatory, antiviral, antioxidant, antibacterial and other effects [7,8,9]. It has been reported in the literature that the most active compounds of EP are polyphenols, such as the following caffeic acid derivatives: caffeic acid, chlorogenic acid, cynarin, echinacoside and cichoric acid [10].

*Ganoderma lucidum* (*G. lucidum*) has been used as a traditional medicine for thousands of years. Polysaccharides and triterpenoids are the main bioactive compounds of *G. lucidum*, and most studies are based on these two classes of compounds [11,12]. *G. lucidum* has a variety of active components, including proteins, enzymes, amino acids, vitamins, minerals, alkaloids, phenolics, flavonoids, glycoproteins, peptidoglycans, and steroids [13,14]. The related literature shows that the bioactive components in G. *lucidum* have a variety of remarkable effects, including immunomodulator [15], anticancer [16], antiviral [17], antioxidant [18], anti-inflammatory [19], cardiac protective [20], antibacterial [21], radioprotective [22], hepatoprotective [23], antidiabetic [24], antipigmentation [25] and antiandrogen [26].

A previous study has shown that EP root extract can inhibit the release of TNF-α to prevent upper respiratory infection [27]. *Echinacea purpurea* polysaccharide (EPP) can inhibit apoptosis and oxidative stress in both in vitro and in vivo models of oxidative damage [28]. Caffeic acid derivatives, alkylamides, polysaccharides, polyacetylenes, polyenes, flavonoids, and terpenoids have been implicated in these biological activities [29].

The antiviral efficacy of *G. lucidum* has been demonstrated with different ganoderma acids (GA), including GA-A, GA-B, GA-C1, GA-C2, GA-β, GA-T, GA-Q, GA-H, Ganoderma alcohol A, Ganoderma lucidum B, Ganoderma diol and Ganoderma triol. The inhibitory effect of these pharmacologically active compounds has been found in different viral infections, including enterovirus 71, hepatitis virus, dengue virus, respiratory syncytial virus (RSV), herpes simplex virus (HSV), HIV virus, H1N1 virus, H5N1 viruses and coronaviruses [30,31].

Mitochondria are vital organelles in cells that primarily function in cellular respiration, energy production, and the regulation of cellular metabolic processes. Recent studies have shown that mitochondria also play a significant role in the combat of viral infections. Many viral infections can directly or indirectly affect mitochondrial function, leading to abnormalities in cellular energy metabolism and biosynthetic pathways, ultimately affecting antiviral immune responses. On the contrary, normal mitochondrial function can enhance cellular antiviral capabilities [32,33]. Therefore, investigating the role of mitochondria in viral infections and how the modulation of mitochondrial function can enhance antiviral capabilities has become a hot topic in current research.

This study adopts the cell pretreatment mode. The cells were first treated with different samples at different concentrations, then infected with influenza virus, and co-cultured for 24 h to observe the changes in the cells. Literature shows that mitochondria play an important role in antiviral activity. Many viral infections can directly or indirectly affect mitochondrial function. Therefore, this study examined the inhibitory and preventive capabilities of EP and *G. lucidum* extracts on influenza A/B viruses by observing the improvement of influenza virus cell viability in different samples and changes in virus titers. The changes of mitochondrial membrane potential were also observed to confirm the correlation between viral infection and mitochondria.

## 2. Materials and Methods

### 2.1. Cell Line and Viruses

Madin Darby canine kidney-2 cells (MDCK-2, American Type Culture Collection, No.CRL-2936) were cultured as previously described [34]. Cells were grown in Dulbecco’s modified Eagle medium (DMEM, Gibco, containing 10% fetal bovine serum and 1% penicillin/streptomycin) at 37 °C in 5% CO_2_. A/Taiwan/117/96 (H1N1) and B/Taiwan/80141/2008 strains were used, which were isolated from patient specimens in the virus room of the Chung Shan Medical University Hospital, stored at −80 °C and quantified in a 50% tissue culture infectious dose (TCID50) test until needed.

### 2.2. Testing Samples

The test samples were provided by Standard Foods Co., Ltd., with a total of three test samples, advanced *G. lucidum* drink (AG), *G. lucidum* extract (GE), and EP extract (EE). The main components of the AG were GE and EE. Samples of GE and EE were extracted with hot water. After solid–liquid phase separation, the supernatant was taken, filtered and packaged. No chemical solvents were used in the whole process. According to the literature, the main possible bioactive components of *Echinacea* and *G. lucidum* are polyphenols and polysaccharides, respectively. Therefore, the samples provided by Standard Food Company were tested for total phenolic and polysaccharide content. The test results are shown in Table 1.

### 2.3. Determination of Total Phenolics and Polysaccharide

The phenol–sulfuric acid method is a simple and rapid colorimetric method for the determination of total carbohydrates in samples [35]. This method detects virtually all classes of carbohydrates, including monosaccharides, disaccharides, oligosaccharides, and polysaccharides. In this method, concentrated sulfuric acid breaks down polysaccharides, oligosaccharides, and disaccharides into monosaccharides. Pentose sugars (5-carbon compounds) are then dehydrated to form furfural, and hexose sugars (6-carbon compounds) are dehydrated to form hydroxymethyl furfural. These compounds then react with phenol to produce a golden color. For products with a high hexose content, glucose is often used to create a standard curve and the absorbance is measured at 490 nm.

The samples used in this study were tested for total phenolic content with reference to the Folin–Ciocalteau (FC) colorimetric method [36]. The Folin–Ciocalteau (FC) colorimetric method is based on the chemical reduction of reagents (a mixture of tungsten and molybdenum oxides). The FC method has been adopted as the official procedure for total phenolic content in wine. The products of metal oxide reduction are blue and exhibit broad light absorption with a maximum absorption wavelength of 765 nm. The intensity of light absorption at this wavelength is proportional to the concentration of total phenols.

### 2.4. HPLC Analysis of Phenolic Compounds

Chromatographic conditions for the analysis of phenolics were adapted from [37]. The analytical column was a SuperSpher1 100, RP-18 cartridge (75 × 4.6 mm i.d.; 3 mm; BDH, Toronto, ON, Canada) with a SuperSpher 100, RP-18 guard cartridge (4 × 4.6 mm i.d.; 5 mm). The mobile phase consisted of 50 mm sodium dihydrogen phosphate adjusted to pH 2.80 with phosphoric acid (solvent A), and 1% 0.1 M phosphoric acid in acetonitrile (solvent B). The elution profile was a linear gradient of 5% to 25% B in 7 min, held at 25% for 2 min, 25% to 5% B in 1 min, and a 5 min equilibration with 5% B: the flow rate was 1.5 mL/min with detection at 320 nm.

### 2.5. Calibration Curve and Quantification

Standard stock solutions of each caffeic acid derivative were prepared. A calibration curve was constructed on five data points covering the concentration range. Each calibration curve was obtained by plotting the peak area of the phenolic compound at each level prepared versus the concentration of the sample. The obtained formula was used to calculate the concentration of each caffeic acid derivative in the sample (results presented in mg/g dry weight ±5%). The quantitative results are shown in Table 2.

### 2.6. Cytotoxicity Test

The cytotoxicity test was evaluated using the previously described SRB assay [38]. The methods and conditions referred to the previous study, with some modifications [39]. Confluent cells were prepared in a 96-well plate with culture medium containing 10% fetal bovine serum. The testing samples were made with two-fold dilution from stock concentration. For the cell control group, 100 μL of growth medium were added into the corresponding wells for sextuplicate. For the treatment group, 100 μL of different concentration of samples were added into the corresponding wells for sextuplicate. Different kinds of cells were cocultured with samples at 37 °C in 5% CO_2_ for 24 h, then replaced with new culture medium. Then, 10 μL of cell Counting Kit-8 (CCK-8) was added to each well. This was mixed thoroughly to achieve a homogeneous solution by lightly tapping the outside of the plate several times while avoiding bubbles. Cells were incubated with a CCK-8 kit for 1 h at 37 °C in 5% CO_2_ until the color changed. The 96-well plate was placed on a shaking table for approximately 1min before the micrometer was read to ensure a uniform color of the orifice plate. The absorbance value (OD) at 450 nm was measured with an enzyme immunoassay analyzer (ELISA reader) and the results were interpreted a reference wavelength of 650 nm [40]. The cell control group was used as a reference value, and the average absorbance value of each group was converted into a percentage.

### 2.7. 50% Tissue Culture Infectious Dose, TCID_50_

The TCID50 assay is an example of an end-point dilution assay that determines the point at which 50% of cells in a culture are infected. In the serum bottle, the virus solution was prepared with serially ten-fold dilution with virus proliferation culture medium, from 10^−1^ to 10^−8^ for the following steps. Confluent cells were prepared in a 96-well plate with culture medium containing 10% fetal bovine serum. In the TCID50 assay method, each dilution of virus solution was added into the corresponding wells for octuplicate. After a suitable infection time, the wells are observed and scored for the presence or absence of cytopathic effect (CPE) (but not quantity). Calculations (or graphs) were then used to plot the percentage of infected cells at each dilution, and the virus dilution at which 50% of the wells had been infected is determined. The calculation used to establish the 50% point was based on that described by [41].

### 2.8. Cell Survival Assay

In this experiment, cells were cultured in a 96-well plate in a medium containing 10% fetal bovine serum. Test samples were prepared by diluting twice the concentration of the original solution. Each set was designed with six replicates. For the cell control group, each well of cells was added to 100 μL of growth medium. For the virus control, cells were treated with 100 TCID50/100 µL virus solution and cultured at 37 °C in 5% CO_2_ for 48 h. The CCK-8 kit was used to interact with living cells, an enzyme immunoassay analyzer (ELISA reader) was used to measure the absorbance value (OD) at 450 nm, and the results were interpreted with 650 nm as a reference wavelength [40]. Using the cell control group as the reference value, the average absorbance value of each group was converted into a percentage.

### 2.9. Hemagglutination Assay (HA Assay)

The hemagglutination assay was developed in 1941–1942 as a method to quantify relative concentrations of viruses, bacteria, or antibodies [42]. The HA assay is based on the ability of certain viral surface proteins to bind to the red blood cells (RBCs) of certain animals. In this experiment, human type O blood was collected and stored in centrifuge tubes containing anticoagulants. Red blood cells and serum were separated using a centrifuge (1500 rpm, 5 min), the serum was removed, and the cells washed three times with PBS at pH 7.2. We diluted with PBS to prepare a 1% red blood cell suspension which was stored at 4 °C. We collected the cell suspension of the cell survival assay, made two-fold serial dilutions with PBS, reacted them with 1% RBC suspension for 1 h, and observed which dilution factor changed from a positive result to a negative result. The results show that the red blood cells are agglutinated and distributed at the bottom of the hole, and that the result is judged as positive; however, if the red blood cells at the bottom of the hole are agglutinated and smooth, the result is judged as negative.

### 2.10. Analysis of Mitochondria Membrane Potential by JC-1 Staining

The protocol, method and conditions referred to the previous study [43]. Confluent cells were prepared in a 24-well plate with culture medium containing 10% fetal bovine serum. All test samples were made from stock solutions with the same concentration (1 mg/mL), and the improvement of mitochondrial conditions at the same concentration was compared. Cells were treated with different samples for 2 h, then added with the same dilution of virus solution and cocultured in a 37 °C 5% CO_2_ for 48 h. Cells were incubated with assay buffer containing JC-1 and at 37 °C for 30 min. At the end of incubation, cells were washed to remove the staining solution. Fluorescence images were collected using an inverted fluorescence microscope and the results represented by the ratio of average red/green fluorescence intensity. Image J (National Institute of Mental Health, Bethesda, MD, USA) software was used to measure red and green fluorescence in each group.

## 3. Results

### 3.1. Virus

**Figure 1:**
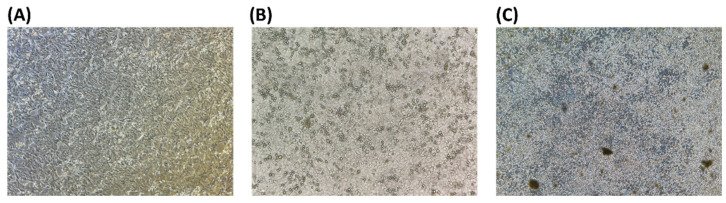
MDCK-2 cells and influenza virus morphology. (**A**) MDCK-2 cells. (**B**) Influenza virus A/Taiwan/117/96 (H1N1). (**C**) Influenza virus B/Taiwan/80141/2008; 40×.

The influenza viruses A/Taiwan/117/96 (H1N1) and B/Taiwan/80141/2008 were proliferated by MDCK-2 cells. The cytopathic effect (CPE) was observed and photographed with a microscope. This confirmed that MDCK-2 cells could be infected to produce CPE (Figure 1).

### 3.2. HPLC Chromatograms of Caffeic Acid Derivatives

**Figure 2:**
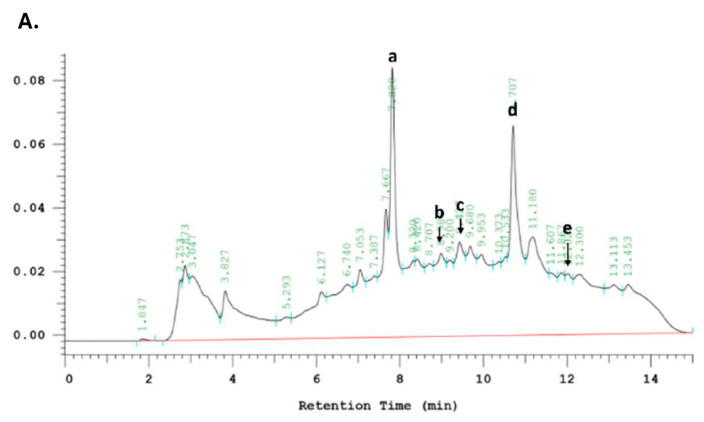

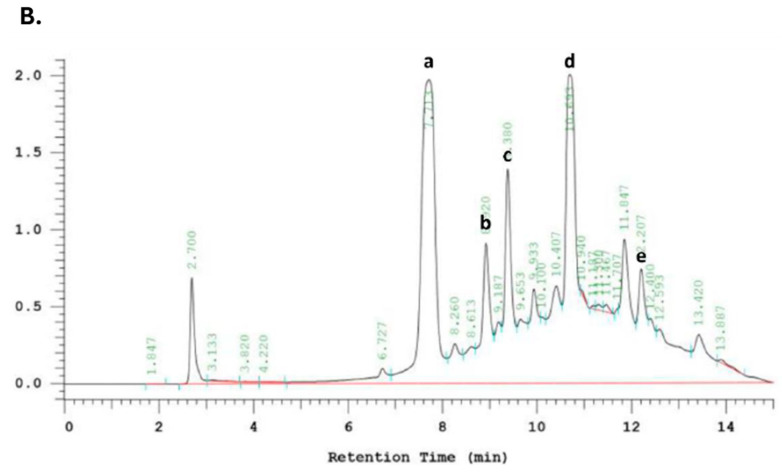
HPLC chromatograms of phenolic compounds of samples. (**A**) Advanced G. lucidu drink (AG). (**B**) Echinacea extract (EE). Detected at absorbance value 320 nm. (**a**) Chlorogenic acid. (**b**) Echinacoside. (**c**) Cichoric acid. (**d**) Caffeic acid. (**e**) Cynarin.

Figure 2 shows the chromatogram of phenolic compounds derived from advanced *G. lucidum* drink (AG) and echinacea extract (EE) detected at 320 nm. Caffeic acid derivatives from *echinacea* spp. have been linked to these bioactivities [44]. The main caffeic acid derivatives include caftaric acid, chlorogenic acid, caffeic acid, cynarin, echinacoside and cichoric acid. Among these, cichoric acid and echinacoside have been widely studied for their remarkable bioactivity and are assumed to be the active ingredients of *Echinacea* [10]. According to the HPLC analysis results, the AG and EE samples contain some major caffeic acid derivatives. After comparison with standards, it was found that they mainly contain chlorogenic acid, echinacoside, cichoric acid, caffeic acid and cynarin.

### 3.3. Cytotoxicity Test

**Figure 3:**
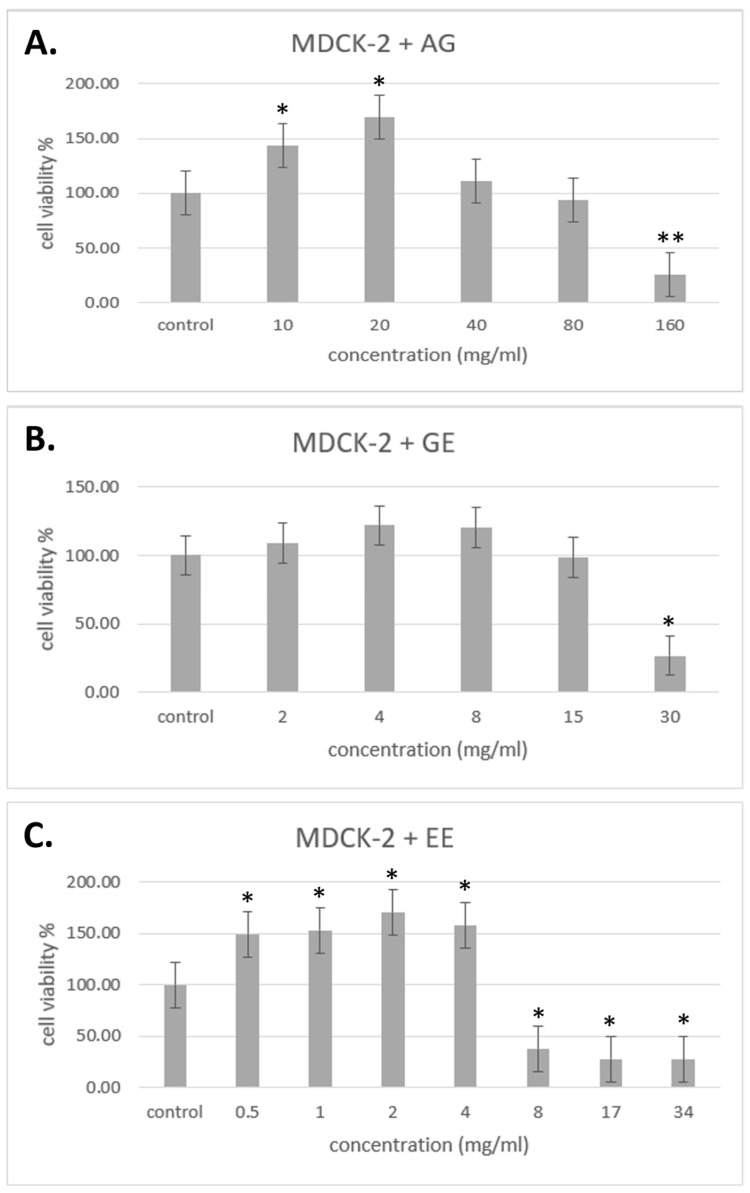
Cytotoxicity test for MDCK-2 cells. (**A**) MDCK-2 cells treated with AG. (**B**) MDCK-2 cells treated with GM. (**C**) MDCK-2 cells treated with EE. Data are shown as mean % relative to control ± S.E.M; * *p* < 0.05 vs. control group; ** *p* < 0.01 vs. control group.

The samples provided by Standard Foods Co., Ltd. (Taoyuan, Taiwan). were used to treat the MDCK-2 cells. Groups treated with different concentrations of samples were converted into percentages with the cell control group as 100%, and the results were determined as noncytotoxic concentrations for those higher than 70%. Figure 3 shows the results of the noncytotoxic concentration of samples in MDCK-2 cells. The noncytotoxic concentration of AG for MDCK-2 cell is lower than 80 mg/mL, GE is lower than 15 mg/mL, and EE is lower than 4 mg/mL. All experiments were performed at noncytotoxic concentrations.

### 3.4. Improvement of Different Samples on Cell Viability with Influenza Virus

**Figure 4:**
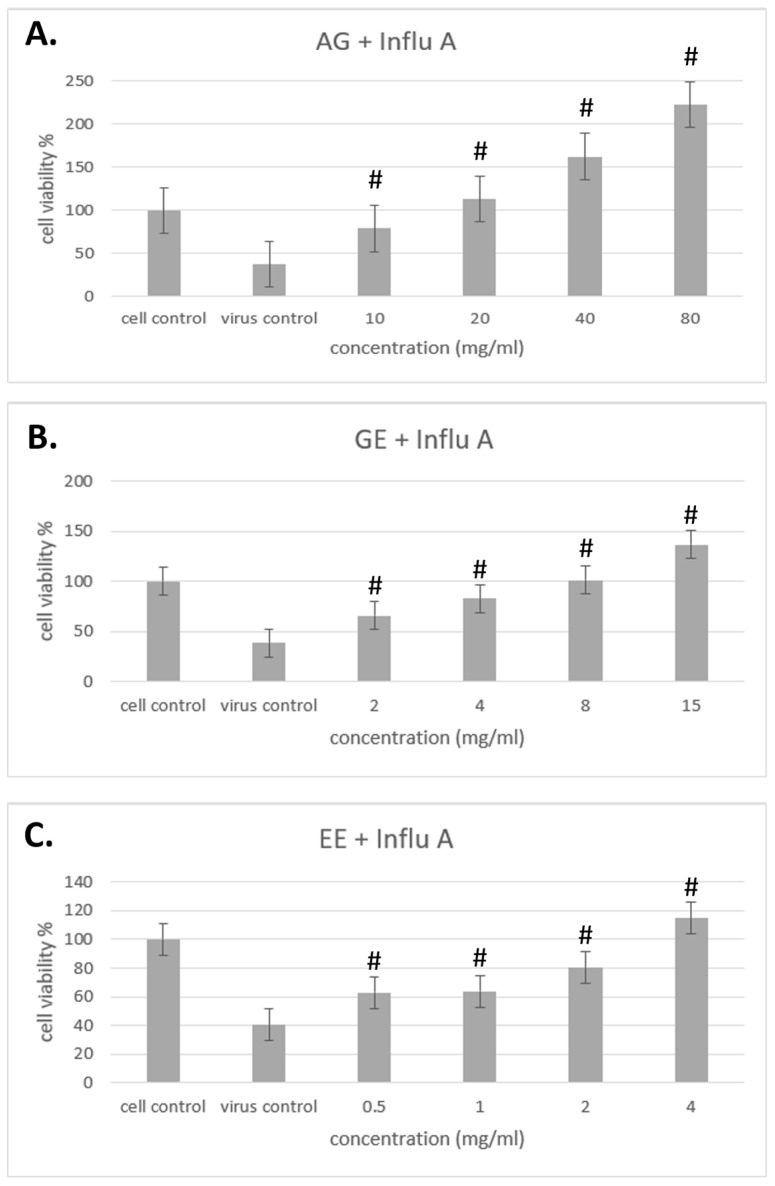
Improvement effect of different samples on cell viability with influenza A virus. (**A**) The effects of different concentrations of AG on influenza A virus cell viability. (**B**) The effects of different concentrations of GM on influenza A virus cell viability. (**C**) The effects of different concentrations of EE on influenza A virus cell viability. Data are shown as mean % relative to cell control ± S.E.M; # *p* < 0.01 vs. virus control group.

**Figure 5:**
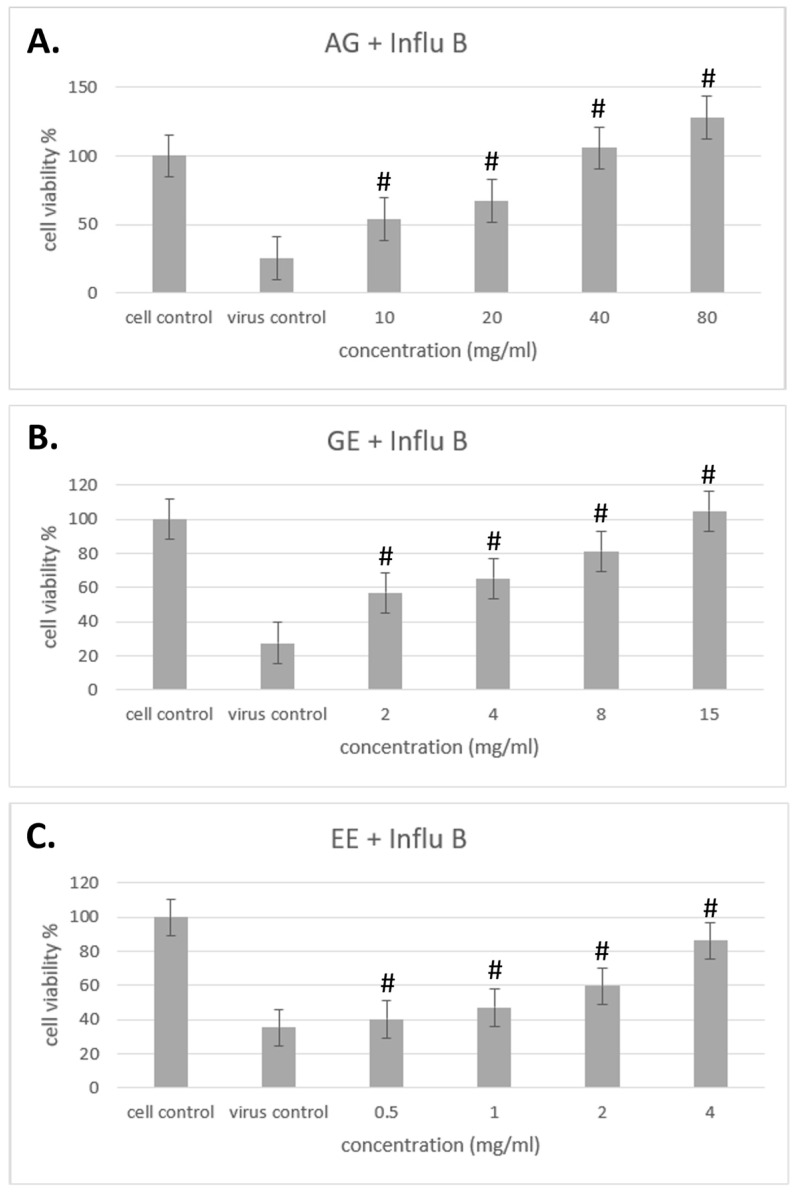
Improvement effect of different samples on cell viability with influenza B virus. (**A**) The effects of different concentrations of AG on influenza B virus cell viability. (**B**) The effects of different concentrations of GM on influenza B virus cell viability. (**C**) The effects of different concentrations of EE on influenza B virus cell viability. Data are shown as mean % relative to cell control ± S.E.M; # *p* < 0.01 vs. virus control group.

The results of different samples against influenza A/B viruses are shown in Figure 4 and Figure 5. In this test, cells were treated with samples for 2 h, then virus solution was added and cocultured for 48 h to observe whether the samples could improve cell viability to prevent influenza A/B infection and achieve preventive effects.

The results show that samples of AG, GE and EE can prevent influenza A virus from invading cells by improving cell viability, and that cell viability increased with increasing concentration, in a dose-dependent manner (Figure 3). The preventive effect of samples on influenza B, all samples can also prevent influenza B virus from invading cells by improving cell viability in a dose-dependent manner (Figure 4). Overall, the main components of AG are GE and EE and, when compared with these, AG itself has a better effect in terms of improving cell viability to prevent influenza A/B infection.

The results of this study are similar to previous studies [44,45,46], *G. lucidum* and EP extracts and mixtures have shown the ability to improve cell viability against influenza A/B viruses. *G. lucidum* and EP extracts and mixtures exhibited the ability to enhance cell survival against viruses. It is speculated that EP and *G. lucidum* extracts can enhance cell immunity and prevent influenza A/B infection.

### 3.5. Hemagglutination Assay (HA Assay)

**Figure 6:**
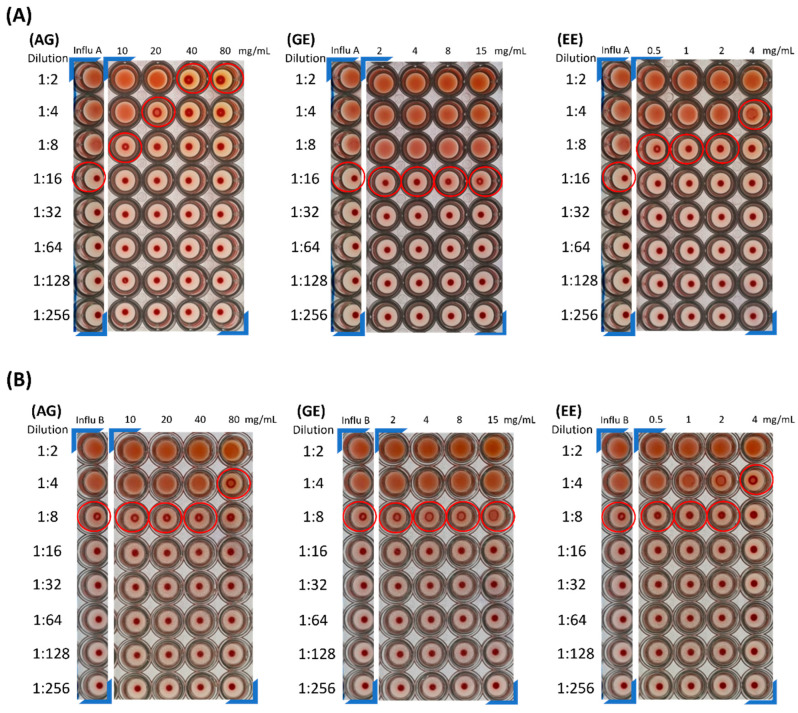
Hemagglutination assay for samples with influenza viruses. (**A**) Titration of samples treated with influenza A virus solutions by hemagglutination assay. (**B**) As in (**A**), titration of samples treated with influenza B virus solutions by hemagglutination assay. The wells denoted by red circles represent the highest dilution to exhibit hemagglutination. This dilution corresponds with HA titer.

From the cell survival assay results, we found that AG, GE and EE exhibited the ability to enhance cell survival against influenza A/B. To confirm whether the samples can directly inhibit the virus, a solution of the highest concentration of virus-containing samples in the cell survival assay was collected, and HA assay was used to observe the effect of EP and *G. lucidum* extracts on direct suppression of the virus. To carry out an HA assay, a two-fold serial dilution of virus-containing samples was dispensed into individual wells of a 96-well microtiter plate (Figure 6). Then, aliquots of RBC were added to each well. The highest dilution at which clumping was observed is regarded as the HA titer of the sample. In a standard condition, 1 HA unit corresponds to 10^4^ particles per mL.

The results show that AG (all concentrations) and EE (4 mg/mL) had a higher inhibition factor on influenza A/B virus when compared with influenza A/B stock solution. EE was found to directly inhibit virus titer with one less HA unit than the influenza A/B stock solution. When the concentration of AG reaches 80 mg/mL, 3 HA units can be reduced, and it still has the ability to reduce the influenza A virus titer as the concentration decreases. However, for influenza B, only when the AG concentration reaches 80 mg/mL can the virus titer be reduced. It was found that GE does not have the ability to directly reduce virus titer. This indicates that the EP extract had a good inhibitory effect on both influenza A/B viruses; AG, whose main components are GE and EE, has a stronger ability to reduce the titer of influenza virus A/B. Previous studies have demonstrated that Echinaforce^®^, an EP extract, broadly inhibits the infectivity of influenza A/B, RSV, parainfluenza virus, and herpes simplex virus in vitro and interferes with cytokine production induced after viral infection [44,47,48]. It is similar to the effect of EP extract on inhibiting influenza virus directly in this study.

### 3.6. Improvement of Mitochondria Dysfunction by Samples

**Figure 7:**
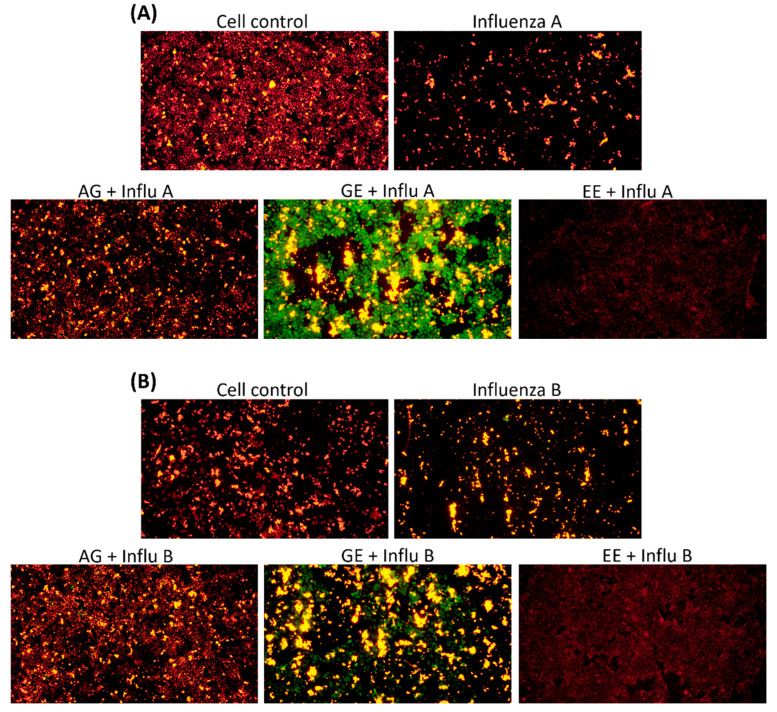

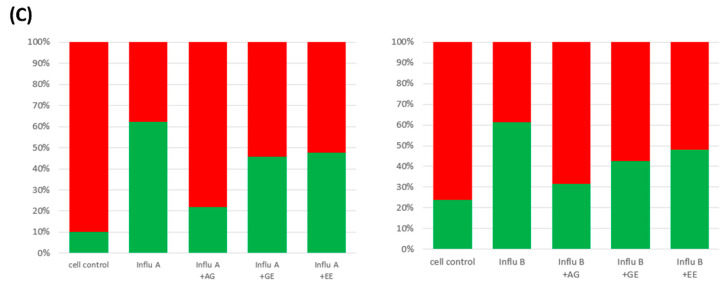
Double fluorescence staining of mitochondrial membrane potential by JC-1. The concentrations of all samples were adjusted to be consistent (1 mg/mL). Green fluorescence indicates the decreased membrane potential, red fluorescence indicates that samples effectively preserve the mitochondrial membrane potential. (**A**) Mitochondrial morphology in cells was stained with JC-1. Samples in same concentration and treated with influenza A virus after 48 hrs. (**B**) As in (**A**), treated with influenza B virus. (**C**) Ratio of red and green fluorescence. Left shows cells treated with influenza A, and the right shows cells treated with influenza B.

Figure 7 shows the changes in mitochondrial membrane potential of samples protected against influenza A/B virus infection. In this experiment, cells were treated with different samples for 2 h, then virus solutions of the same dilution were added and cocultured for 48 h at 37 °C and 5% CO_2_ in order to explore whether the samples have the effect of improving mitochondria to prevent influenza A/B virus infection in vitro. To compare the effects of all samples in terms of their improvement of the mitochondrial membrane potential, the concentrations of all samples were adjusted to be consistent (1 mg/mL). As shown in Figure 6, influenza A/B virus treatment resulted in a strong increase in green fluorescence, indicating a great loss of mitochondrial membrane potential by influenza A/B virus. The results show that treatment with samples at the same concentration can decrease the green fluorescence, suggesting that the EP and *G. lucidum* extracts preserve mitochondrial function against influenza A/B virus-induced mitochondrial dysfunction, and that AG has a better improvement effect.

## 4. Discussion

The antiviral potential of *G. lucidum* extract has also been a focus if this study and has been elucidated upon, showing that it has complex components and multiple biological activities [49]. The ganoderic acid (GA) in *G. lucidum* exhibits potential effects against the influenza virus. Inhibition is manifested in the inhibition of the neuraminidase enzyme responsible for the release of the influenza virus from host cells. Inhibition occurs through interactions with amino acid residues Arg292 or/and Glu 119 of the enzyme [46]. Fungal immunomodulatory proteins (FIPs) in *G. lucidum* have been reported to have a significant effect on the inhibition of respiratory syncytial virus (RSV). Furthermore, the bioactive protein isolated from other mushrooms has been shown to effectively reduce RSV replication, inflammation, and IL-6 expression by inhibiting NF-κB translocation inhibition [20,45]. Previous studies testing rhinovirus 1A/14, influenza virus, RSV, adenovirus types 3 and 11, and herpes simplex virus type 1 evaluated the ability of the ethanol extracts of EP root part to modulate viral induction of cytokines. EP inhibits this induction, showing antiviral potential [44]. Previous studies have demonstrated the direct antiviral activity of EP extract against common cold coronavirus 229E (HCoV-229E) and highly pathogenic coronaviruses (SARS-CoV-1 and MERS-CoV) [50].

In the HA assay, each group was tested using a separate 96-well plate and independently compared with the virus control group to ensure that the conditions and the virus control group were consistent when conducting the experiment. The HA assay mainly measures whether the sample can directly inhibit the titer of influenza virus. The first step of influenza virus entry into cells depends on the interaction between viral HA and specific cellular sialic acid receptors. If this interaction can be inhibited by binding to HA, it may prevent viral entry. Receptor binding of functional HA can be measured by its ability to agglutinate erythrocytes, which can be easily counted visually [42]. However, inhibition required direct contact between the virus and the sample, as pretreatment of the cells prior to virus infection, or exposure of the cells to the sample, resulted in a marked reduction in inhibition, indicating that the antiviral effect was at an early stage in the infection process. In summary, EP extract has a good inhibitory effect on influenza A/B and can significantly improve cell viability in the results of antiviral activity tests. The mixture of *G. lucidum* and EP extracts did not have better anti-influenza virus ability. This was confirmed using an HA assay, which clearly showed that the EP extract inhibited HA activity and thus prevented the entry of the treated virus into the cells. However, the mechanism of inhibition needs to be investigated in more experiments. HA assay is a classical method for viral diagnosis, still used for diagnosis of influenza virus today. One outstanding advantage of this method is that it does not require any equipment. Moreover, it is a robust and rapid diagnostic tool, though the sensitivity is somewhat limited [51].

Among caffeic acid derivatives, only cichoric acid exhibits immunostimulatory properties, promoting phagocyte activity in vitro and in vivo [52]. In addition, cichoric acid has anti-hyaluronidase activity [53]. Cichoric acid also has antiviral activity [54] and was found to inhibit HIV-1 integrase and replication [55]. Echinacoside does not have immunostimulatory activity, but it can protect collagen from the effects of reactive oxygen species [56] and has antioxidant activities [57]. Studies have found that cichoric acid is the main phenolic compound in echinacea. Echinacoside is the major phenolic component in the roots of *E. angustifolia* and *E. pallida*. However, according to the HPLC results of this study, echinacea extract contains cichoric acid and echinaccoside, though in small amounts. Some of the antiviral activity was provided by cichoric acid, which previous studies have demonstrated to be moderately active against HSV [58], and other caffeic acid derivatives that may be present in these fractions. However, there was no clear correlation between phenolic concentration and relative activity, suggesting the presence of additional, perhaps more potent, antiviral compounds.

Caffeic acid (3,4-dihydroxycinnamic acid) is a rich, plant-based polyphenolic compound with 2 phenolic hydroxyl groups. This compound is the main metabolite produced by the hydrolysis of chlorogenic acid. A previous study has demonstrated that caffeic acid effectively inhibits the proliferation of influenza A viruses, and similar to the results of this study. This may be due to the specific interaction of caffeic acid with the cellular and/or viral proteins involved during viral genome replication [59].

Cynarin, 1,3-dicaffeoylquinic acid, is formed from the esterification of two units of caffeic acid and one unit of quinic acid. It has been shown to have some pharmacological properties, including hypocholesterolemic [60], hepatoprotective [61], antiviral, antibacterial, and antihistamic effects [62]. The isolation of cynarin from natural sources has been reported, but yields are low and the purity is poor. Studies have shown that, in in vitro bioassays, cynarin was found to be a powerful antioxidant, antiradical, and anticholinergic agent that protects the body from the effects of free radicals and reactive oxygen species [63].

## 5. Conclusions

For respiratory infection viruses, vaccination can be used to prevent infection or drug treatment can be used to reduce symptoms after infection. However, after infection, some symptoms persist, a phenomenon commonly known as “long COVID”. This indicates that there has been harm done to the human body. In this study, it is speculated that mitochondria play a potentially important role after infection with influenza virus. The results of this study indicate the ability of EP and *G. lucidum* extracts to resist the influenza A/B viruses by preventing virus entry into cells, improving cell viability and mitochondrial membrane potential. The results also indicate that AG and EE directly decrease influenza virus titers. The results of this study provide a correlation between the preventive effect of *G. lucidum* and EP extract and cell function. By improving cell function, influenza virus infection can be prevented and mitochondrial damage can be improved. COVID-19, which has been prevalent since 2019, is mainly infected through the human respiratory tract. To this day, many mutant strains have emerged, making the virus spread faster, and there are even examples of long COVID, in which the virus damages the body and causes symptoms to persist for weeks or even years after recovery. According to the results of this study, EP has the ability to directly inhibit viruses, enhance cell performance, and improve the performance of mitochondrial membrane potential. To confirm this indication, cell models will be used in future studies to simulate infection with upper respiratory infection viruses in order to observe the changes of mitochondria in cells and to explore the role of mitochondria in antiviral efficacy.

## Figures and Tables

**Table 1:** Content of polysaccharide and total phenolic compounds in analyzed extracts.

Samples	Content (mg/g Dry Weight)	
	Polysaccharide	Total Phenolic
AG	30.23 ± 1.51 ^a^	0.28 ± 0.01
GE	145.16 ± 7.258	-
EE	-	386.98 ± 19.35

^a^ SE—standard error.

**Table 2:** Quantification of caffeic acid derivatives in samples.

	AG	EE
	mg/g Dry Weight	
Chlorogenic acid	0.28 ± 0.01	131.15 ± 6.56
Echinacoside	0.30 ± 0.01	37.03 ± 1.85
Cichoric acid	0.13 ± 0.06	40.26 ± 2.01
Caffeic acid	0.53 ± 0.03	261.48 ± 13.07
Cynarin	0.04 ± 0.002	14.50 ± 0.72

## Data Availability

Data are contained within the article materials.
